# Genetic characterization on the nucleoprotein and fusion gene of wild-type measles virus circulating in Shanghai, 2001–2022

**DOI:** 10.1016/j.jve.2025.100589

**Published:** 2025-03-05

**Authors:** Yunyi Li, Xiaoxian Cui, Ai Lin, Wei Tang, Yuying Yang, Wanju Zhang, Jiayu Hu, Zhi Li, Yanqiu Zhou

**Affiliations:** a Institute of Microbiology Laboratory, Shanghai Municipal Center for Disease Control and Prevention, Shanghai, 201106, China; b EPI Department, Shanghai Municipal Center for Disease Control and Prevention, Shanghai, 201106, China; c Institute of Microbiology Laboratory, Shanghai Institute of Preventive Medicine, Shanghai, 201106, China

**Keywords:** Measles, Fusion protein, Measles vaccine, Shanghai-191, Genotyping, Viral surveillance

## Abstract

Measles is an acute and highly contagious viral disease that poses significant public health challenges globally. Since 2001, continuous virologic surveillance has been conducted in Shanghai, enabling a comprehensive analysis of the evolution of the nucleoprotein (N gene) and fusion gene (F gene) of the measles virus (MeV) over a 21-year period. Between 2001 and 2022, there were a total of 1405 MeV strains isolated by the Shanghai Center for Disease Control and prevention (SCDC), including 6 strains of genotype D8, 8 strains of genotype B3, 12 strains of genotype H1b, and the remaining strains of genotype H1a. Reverse transcription polymerase chain reaction (RT-PCR) was used to amplify the 3′ end of the N gene (450 nt) and the complete sequence of the F gene (1622 nt) from the viral isolates. Sequencing of the RT-PCR products was followed by nucleotide and amino acid phylogenetic analyses. The substitution rates were for the F and N genes in Shanghai were determined to be 0.89 × 10^−3^ and 2.20 × 10^−3^ substitutions site^/^year, respectively.

Globally, the nucleotide and amino acid similarities of the N gene among 13,498 MeV isolates ranged from 89.1 %–100.0 % and 90.2 %–100.0 %, respectively. Notably, the F gene exhibited 16 high-amino-acid-mutation sites, most of which differed among H1a MeV strains compared to the Shanghai-191 vaccine strain. The deletion of the glycosylation site at aa 9–11(NVS) was primarily observed in H1a and H1b of MeV strains. However, critical functional sites in the F gene remained conserved.

In conclusion, the previously predominant indigenous H1a wild-type measles virus (MeV) has not been detected for over two years, with only imported MeV genotypes currently being identified. It is crucial to strengthen the surveillance of MeV genotypes to facilitate the timely identification and containment of imported measles cases, thereby preventing potential outbreaks.

## 1 Introduction

Measles is a highly infectious acute febrile and eruptive disease caused by the measles virus (MeV), which belongs to the *Morbillivirus* genus within the *Paramyxoviridae* family. The virus is characterized by a 15,894-nucleotide-long, non-segmented, single-stranded RNA genome with negative polarity. This genome encodes six structural proteins (nuclear protein (N), phosphoprotein (P), membrane protein (M), fusion protein (F), hemagglutinin (HA), and polymerase protein (L)) as well as two non-structural proteins. Among these regions, the 450-nucleotide sequence at the carboxyl-terminal (COOH) end of the nucleoprotein (N) gene exhibits the highest variability and is recommended by the World Health Organization (WHO) as the standard target for molecular genotyping and identification of MeV genotypes.^1^ To date, WHO has recognized 24 genotypes based on N gene sequence analysis, with genotypes H1, D8, D4 and B3)being prevalent globally in recent years.^2^

The F gene, comprising 1662 nucleotides encoding 553 amino acids, is relatively conserved, The F protein plays a crucial role in inducing neutralizing antibodies in the host and is essential for viral membrane fusion with the HA protein at the host cell membrane.^3^ Additionally, the F protein is involved in viral replication, cytopathic effect (CPE), cell tropism, and other biological functions.^4^

Despite the continuous progress of measles elimination plans, the incidence of measles in China has significantly declined since 2014, reaching a historical low between 2020 and 2022.^5^ However, global measles cases have increased annually since 2016, with a notable surge in 2019, reporting 837,922 cases and an incidence rate of per 120 per million, more than fivefold higher than in 2016.^6^ Notably, measles outbreaks have reemerged in the United States and certain European countries, despite having achieved measles elimination status.

This study aims to analyze the phylodynamic features and evolutionary estimates of measles virus isolated from patients in Shanghai over a 21-year timespan by comparing the N gene nucleic acid. The N protein is the most abundant protein in the measles virus particle and plays a crucial role in the virus's transcription and replication. During the acute phase of measles virus infection, it is primarily the N protein that mediates the cellular and humoral immune responses in the human body. Therefore, mutations in the measles virus N protein can affect the immune efficacy of the measles vaccine.^7^ Although recent studies have placed less emphasis on the measles fusion (F) gene, it is noteworthy that mutations within this gene can result in the production of low-molecular-weight F proteins, which have been implicated in the pathogenesis of subacute sclerosing panencephalitis (SSPE), a rare yet severe and fatal neurological complication associated with measles virus infection. Recent advancements in the development of anti-membrane fusion drugs highlight the potential for improved treatment of such conditions. Therefore, by comparing the nucleotide and amino acid differences, as well as glycosylation site changes, between MeV strains and the S191 vaccine strain from 2001 to 2022, this study seeks to elucidate the variation of the F gene in Shanghai, assess its impact on the immunogenicity of the S191 vaccine, and provide foundational data for understanding the molecular mechanism of measles virus-related membrane fusion.

## 2 Materials and methods

### 2.1 MeV surveillance laboratory network

Since 2001, a comprehensive network of measles surveillance laboratories has been established in SCDC and CDC of each district. This network is responsible for isolating measles virus (MeV) from throat swab samples and detecting measles/rubella virus-specific immunoglobulin M (IgM) antibodies in serum using enzyme-linked immunosorbent assays (ELISA).

### 2.2 Specimen collection and transportation

All specimens in this study were collected and transported by district CDCs and hospitals participating in the MRLN in Shanghai. Throat swab specimens were obtained from suspected measles cases, preserved in 2 mL MEM media, and transported to SCDC by a 4 °C cold chain for 24 h for MeV isolation.

Since April 2014, throat swabs were first transported to district CDCs for MeV RNA screening by using real-time PCR before being sent to the SCDC for further isolation of positive specimens. High-quality surveillance of pathogenic measles has been maintained in Shanghai since 2001. Starting in 2011, in accordance with the national measles surveillance program, throat swabs were collected for MeV isolation from every suspected measles case. Since 2014, nucleic acid detection and genotyping of MeV and rubella virus have been conducted systematically.

### 2.3 Measles virus isolation

MeV isolation were performed using Vero/hSLAM cells. Cell maintenance, sample handling, viral infection, and cytopathic effect (CPE) observation were conducted according to the WHO measles manual^8^ and the methodology described by Yanagi et al.^9^

### 2.4 Viral RNA extraction and reverse transcription polymerase chain reaction (RT-PCR

Total RNA was extracted from viral isolates using the MagMAX TM-96 Viral RNA Isolation Kit (Thermo, Vilnius, Lithuania) and KINGFISHER FLEX automatic nucleic acid extraction instrument (Thermo Fischer Scientific, Waltham, MA, USA) following the manufacturer's instructions. The extracted RNA was dissolved in 50 μL of nuclease-free water and stored at −20 °C.

For RT-PCR amplification, the 3′ end of the N gene(450 nt) was amplified using the PrimeScript One-Step RT-PCR Kit (TaKaRa, Liaoning, China).^10^ The complete coding region of the F gene (1662 nt) was amplified using standard primers provided by the U.S. Centers for Disease Control and Prevention (USCDC)^11^: forward primer, 5-‘GAA TCC CAG AAT CAA GAC TCA TC-3’, reverse primer, 5-‘CAT TTG TGT TTC AAG AGT TG-3’. RT-PCR was performed using the SuperScript^TM^ III One-Step RT-PCR System with Platinum®Taq High Fidelity (Thermo Fischer Scientific). The reaction mixture (50 μL) contained 4 μL template RNA, 25 μL of 2X reaction mix, 1 μL High Fidelity Enzyme Mix, 1 μL forward primer (10 μM), 1 μL reverse primer (10 μM), and 18 μL nuclease-free water. The amplification conditions were as follows: reverse transcription at 55 °C for 30 min, initial denaturation at 94 °C for 2min, followed by 40 cycles at 94 °C for 15 s, 50 °C for 30 s, 68 °C for 130 s, and a final step at 68 °C for 5 min. Electrophoresis was performed to separate the amplicons using a 1.5 % agarose gel and gel red (Biotium, CA, USA). Gel images were obtained using a ChemiDoc^TM^ XRS+ (Bio-Rad, CA, USA).

### 2.5 Nucleic acid sequencing and analysis

A total of 1369 N-450 and 358 F gene sequences were obtained from MeV strains isolated in Shanghai between 2001 and 2022. Additionally, 12,129 N gene and 415 F gene sequences were downloaded from Genebank (1956–2022), representing data from 5 continents(Asia, Africa, North America, Europe, and Oceania). The final datasets comprised 13,498 N gene sequences and 773 F gene sequences, respectively.

Nucleotide and deduced amino acid sequences were aligned using BioEdit software (version 7.0).^12^ Phylogenetic trees were constructed using Mega 6^13^ with the maximum likelihood method (ML), and 1000 bootstrap replicates.

### 2.6 Bayesian Markov chain Monte Carlo (MCMC) evolutionary analysis

For Bayesian skyline plots were curated by removing highly similar MeV strains (less than 1 % nucleotide divergence) from Shanghai (2001–2022). A total of 171 N gene and 191 F gene sequences were selected based on temporal distribution and nucleotide diversity. The general time reversible (GTR) model with a gamma distribution (GTR + G) was determined as the best-fit nucleotide substitution model using ModelTest 2.1.10.^14^ Bayesian MCMC analysis was conducted in BEAST (v1.7.2)^15^ with an uncorrelated exponential relaxed clock model. The analysis was run for 20 million states, sampling every 20,000 states, ensuring a sufficient sample size (n > 200) for each parameter. Bayesian skyline plots were generated using TRACER (v1.5) to depict relative viral genetic diversity over time for the F gene.

## 3 Results

### 3.1 Genotype distribution and analysis of nucleotide and amino acid similarity of MeV in Shanghai (2001–2022)

From 2001 to 2022, a total of 1405 throat swab samples were cultured for MeV isolation (Supplementary Table 1). Phylogenetic analysis revealed that the MeV strains isolated in Shanghai belonged to four main genotypes: H1a (1379/1405, 98.15 %), H1b (12/1405, 0.85 %), D8 (6/1405, 0.43 %), and B3 (8/1405, 0.57 %). The H1a genotype has remained the dominant strain in Shanghai throughout this period. The first occurrences of D8 and B3 were detected in 2012 and 2013, respectively.^16^ These genotypes were sporadically detected in subsequent years: B3 in 2014, 2016, 2018, and 2019, as well as D8 in 2014, 2016, and 2019.^17^

**Fig. 1:**
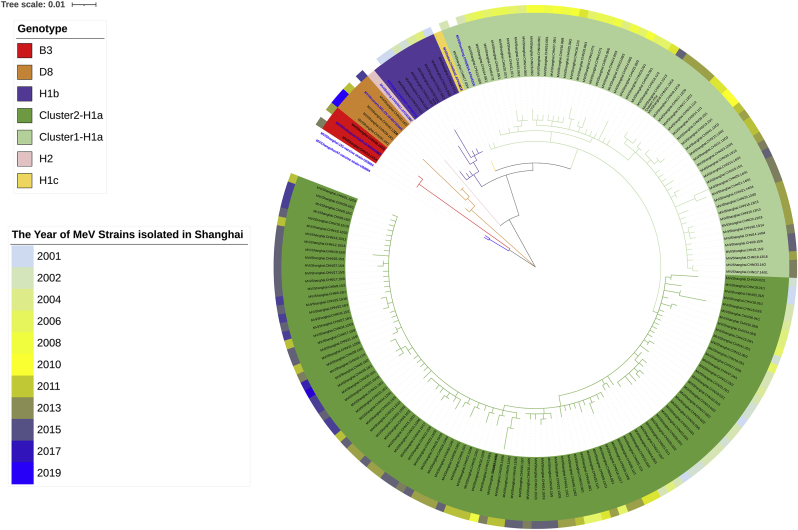
Phylogenetic tree of N gene in Shanghai.

To further analyze the genetic diversity, the N gene of 171 strains isolated in Shanghai (2001–2022) was examined by constructing phylogenetic trees with reference strains, including the S191 and C47 vaccine strains from GenBank (Fig. 1). The nucleotide and amino acid similarities of the N gene sequences from these strains ranged from 88.4 % to 99.8 % and 83.9 %–100 %, respectively. When compared to the S191 vaccine strain, the nucleotide similarities ranged from 91.1 % to 94.4 %, while amino acid similarities ranged from 85.2 % to 91.9 %.

**Fig. 2:**
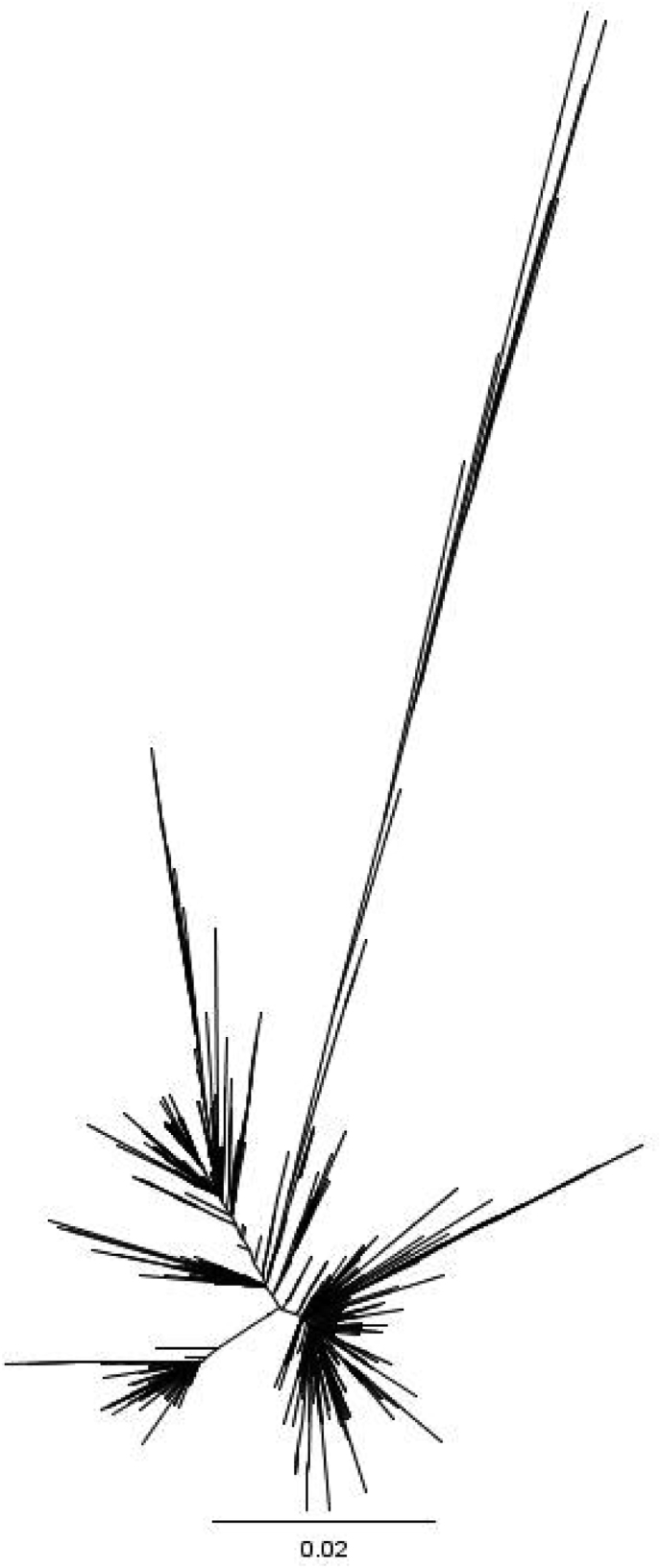
Global phylogenetic tree of N gene for H1a.

A global phylogenetic tree was also constructed by comparing the N gene sequences from 13,498 strains worldwide (1956–2022) with the 1369 strains isolated in Shanghai (Fig. 2). This global analysis showed that the most prevalent genotypes were D8 (4,797, 35.5 %), B3 (3,320, 24.6 %), and H1a (3,047, 22.6 %). In China, H1a remains the dominant strain, representing 90.6 % of the isolates (2759 out of 3047).

### 3.2 Analysis of amino acid mutation in the N gene and F gene

Based on the amino acid mutation analysis of the 150 amino acids (aa376-525) at the carboxyl terminus of the N gene, there are 9 common amino acid substitutions between Cluster 1(2001–2015), Cluster 2 (2001–2018) of the Shanghai H1a strain and the S191 vaccine strain. There are 3 amino acid differences between Cluster 1 and Cluster 2. Compared with the S191 vaccine strain, the B3 strain shows new amino acid substitutions at 10 amino acid sites in the N gene: aa427, 443, 448, 451, 455, 462, 467, 473, 481, 509. The D8 strain shows new amino acid substitutions at 10 amino acid sites in the N gene compared with S191: aa406, 431, 441, 451, 456, 470, 473, 482, 505, 509 (Table 1).

**Table 1:** Amino acid substitution analysis of Shanghai Wild-Type MeV strains and S191 strains.

Strains	AA Position of N Protein																							
	406	422	427	431	441	443	447	448	450	451	455	456	457	462	467	470	471	473	481	482	484	497	505	509
S191/A	I	G	N	R	K	S	A	R	S	Y	G	P	S	A	L	G	T	P	S	S	D	R	S	G
Cluster1-H1a	T	S	N	G	K	S	T	R	N	S	G	P	S	A	L	S	T	P	Y	S	E	R	L	G
Cluster2-H1a	T	G	N	G	K	S	T	R	N	S	G	P	G	A	L	S	T	P	Y	S	E	K	L	G
B3	I	G	S	R	K	N	A	G	S	H	E	P	S	V	P	G	T	L	F	S	D	R	S	D
D8	T	G	N	G	R	S	A	R	S	N	G	S	S	A	L	S	T	L	S	G	D	R	L	S

**Fig. 3:**
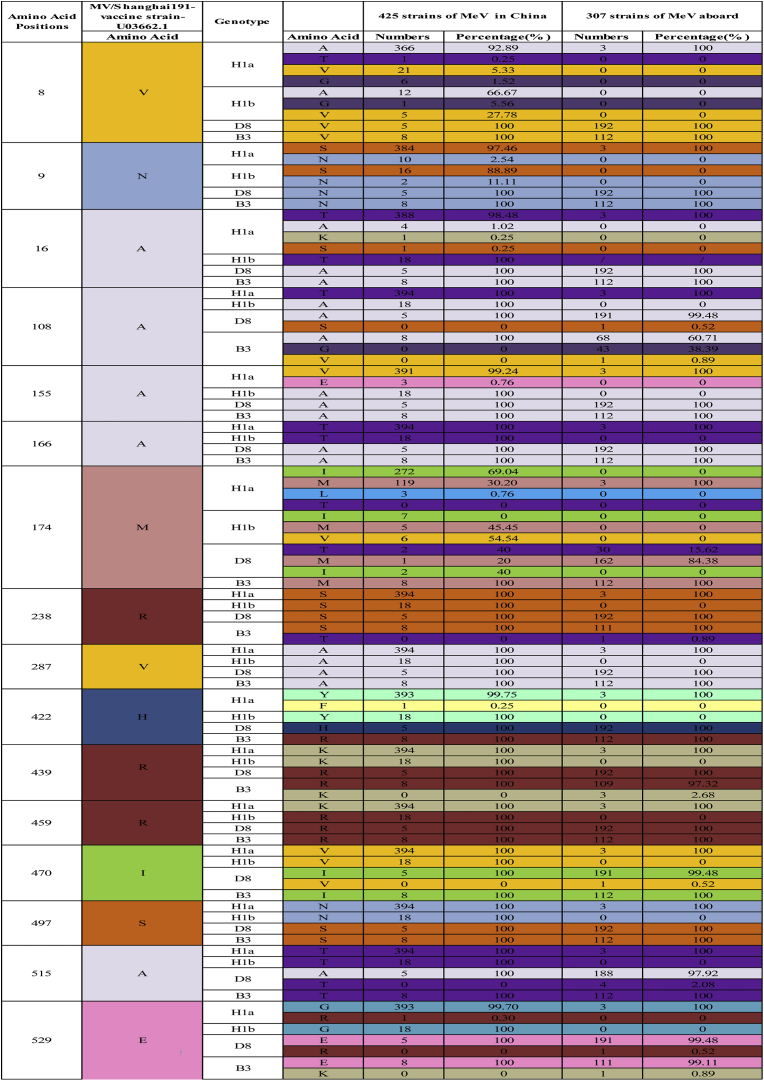

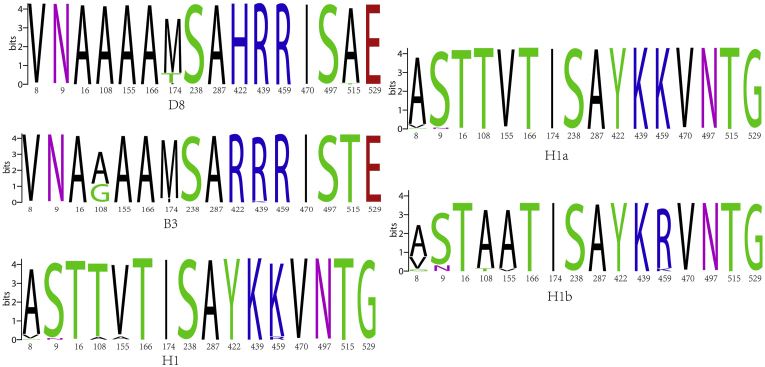

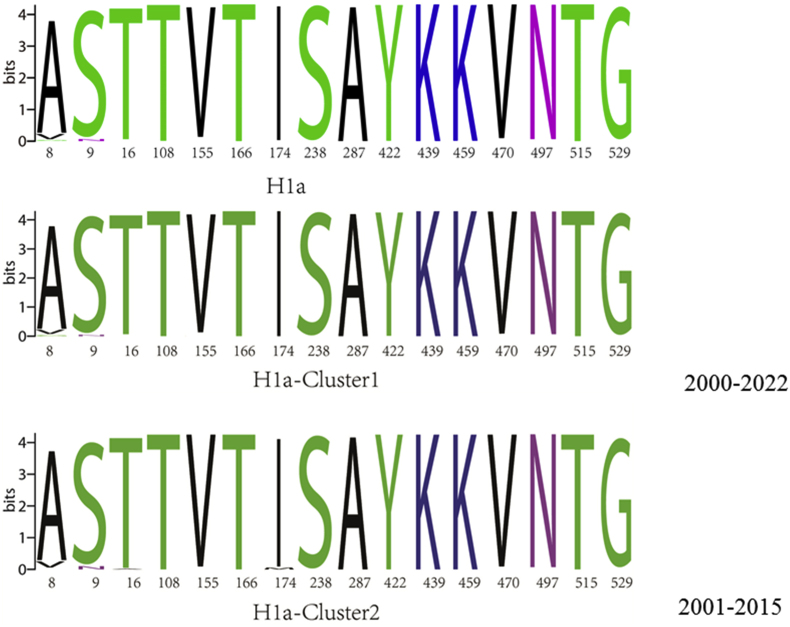
Diversity of Amino Acid of F Gene of MeVs in China and abroad compared with S191Vaccine strain(**A**) 16 high amino acid mutation sites in F protein for different MeV sub-genotypes(**B**) 16 high amino acid mutation sites in F protein in two clusters for H1a (**C**).

By comparing the amino acid sequences of the F protein in 425H1a, H1b, D8 and B3 strains of MeV in China and 307 strains from aboard with the S191 vaccine strain, we identified 16 high amino acid mutation sites (8, 9, 16, 108, 155, 166, 174, 238, 287, 422, 439, 459, 470, 497, 515, 529) in sub-genotype H1a (Fig. 3A). Sub-genotype D8 exhibited only 4 high amino acid mutation sites (1, 11, 238, 287), while sub-genotype B3 had 13 (1, 10, 13, 19, 25, 108, 142, 238, 287, 422, 515, 522, 525). All D8 and B3 strains had an M→V at aa1, altering the promoter codon. The F gene contains four glycosylation sites: aa 9–11(NVS), aa 32–34(NLS), aa 64–66(NIT), and aa 70–72(NCT). (97.5 %, 387/397)Strains of H1a and (88.9 %, 16/18)strains of H1b had an N→S at aa9 mutation, resulting in the deletion of the glycosylation site at aa 9–11(NVS), whereas strains of D8, and B3, showed no change in the Asn9. For aa108, 100 % (397/397) of H1a strains had an A→T mutation, while 100 % (18/18) of H1b, 63.3 % (76/120) of B3, and 99.5 % (196/197) of D8 strains showed no change. In 2019, 36.7 % (44/120) of B3 strains from Korea exhibited an A→G mutation at aa108. Arg112, Gln195 and His419, critical sites, remained conserved (Fig. 3B). Both clusters of H1a for F gene exhibited consistent high amino acid mutation rates (Fig. 3C).

### 3.3 Analysis of evolution in N and F genes

BEAST software was used to calculate the mean nucleotide substitution rate for N and F genes. The mean substitution rate for gene F was estimated at 0.89 × 10^−3^ substitutions site/year (95 % HPD, 0.71 × 10^−3^-1.08 × 10^−3^), whereas the substitution rate for gene N was estimated to be approximately twice as high, 2.20 × 10^−3^ substitutions site/year (95 % HPD of 1.56 × 10^−3^-2.91 × 10^−3^).

**Fig. 4:**
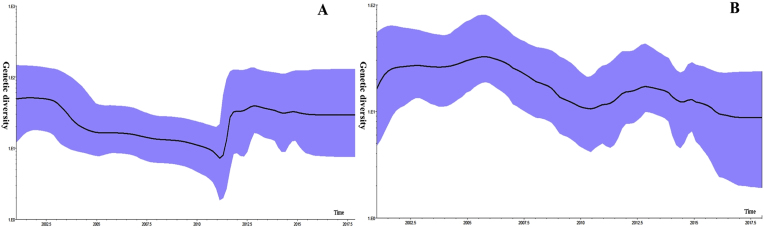
Bayesian skyline plots shows diversity for (**A**) N and (**B**) F genes of representative strains of measles virus isolated from 2001–2019 in Shanghai.

A Bayesian skyline plot for the N gene revealed three distinct phases from 2001 to 2019 in Shanghai. From 2001 to 2010, minimal changes in genetic diversity were observed owing to the limited data availability. From 2011 to 2013, the in genetic diversity increased significantly, correlating with a rise in confirmed measles cases. A stable trend was observed between 2013 and 2019 (Fig. 4A).

For the F gene, the Bayesian skyline plot remained stable from 2001 to 2019 in Shanghai. A slight decline was observed between 2001 and 2010. From 2011 to 2013, genetic diversity increased similarly to the N gene, followed by a slight decrease between 2013 and 2019. Overall, the mutation rate for the F gene was slightly lower than that of the N gene (Fig. 4B).

## 4 Discussion

The significant reduction in measles incidence in Shanghai, nearing the goal of elimination, is a testament to China's comprehensive public health strategies, including high vaccination coverage, robust epidemiological surveillance, and effective outbreak response measures. However, the cyclical nature of measles epidemics and the disruptions caused by the COVID-19 pandemic necessitate continued vigilance against potential measles resurgence. Ongoing virological research on measles virus (MeV) is crucial for providing scientific evidence to support measles prevention, control, and elimination efforts.

The marked decline in measles incidence in Shanghai, approaching the elimination target, underscores the success of China's comprehensive public health strategies, which include high vaccination coverage, robust epidemiological surveillance systems, and effective outbreak response mechanisms. Nevertheless, the cyclical pattern of measles epidemics, compounded by disruptions caused by the COVID-19 pandemic, highlights the need for sustained vigilance to mitigate the risk of measles resurgence. Continued virological research on measles virus (MeV) remains essential to generate scientific evidence that informs and strengthens measles prevention, control, and elimination efforts.

From 2001 to 2022, the native H1a genotype has not been detected since 2019. In recent years, the imported genotypes can be detected in a timely manner, which provides a virological basis for the improvement of laboratory monitoring indicators and the revision of monitoring strategies for measles elimination in China.

The N gene being the most variable region in the measles genome, plays a critical role in immune response and vaccine efficacy. Our study revealed that nucleotide and amino acid similarities were lower for the N gene compared to the fusion (F) gene, consistent with previous findings.^18^ The evolutionary rates for the F and N genes were 0.89 and 2.06 × 10^−3^ substitution site^/^year, respectively. The F gene exhibits a relatively conserved genetic diversity compared to the N gene, reflecting its evolutionary stability. While the observed mutation rates for the F gene are consistent with those reported in Song et al.'s study, discrepancies in the N gene mutation rates are likely attributable to variations in the datasets analyzed, such as the inclusion of multiple genotypes in the present study.^19^

The carboxyl - terminal region (aa 401–525) of the N protein represents a naturally unfolded structural protein that protrudes from the surface of the nucleoprotein. Within this region, there exists a viral polymerase - binding epitope (aa 459–507), where the molecular recognition epitope (aa 488–499) plays a crucial role in the binding process with the phosphoprotein. In the viral polymerase - binding epitope region, distinct amino acid mutations are observed across different genotypes and strains. Specifically, in the B3 genotype, amino acid mutations occur at aa 462, 467, 473, and 481. The D8 genotype exhibits mutations at aa 470, 473, 482, and 505. For the Cluster1-H1a strains, mutations are found at aa 470, 481, 484, and 505. Meanwhile, the Cluster2-H1a shows mutations at aa 470, 481, 484, 497, and 505. In the molecular recognition epitope (aa 488–499), only the Cluster2-H1a strains display a substitution of lysine (K) for arginine (R) at aa 497. Whether these mutations will have an impact on the synthesis of the phosphoprotein within this region and influence its pathogenicity remains to be determined through further research.

The F gene, which plays a critical role in viral membrane fusion, demonstrated 16 high-amino-acid-mutation sites in the H1a sub-genotype. Notably, all D8 and B3 strains exhibited an M→V mutation at aa1, potentially altering the promoter codon and affecting viral replication. Moreover, the N→S mutation at aa9, leading to the deletion of the glycosylation site (NVS), was commonly observed in H1a and H1b strains but absent in D8 and B3 strains. Although the functional impact of this deletion remains unclear, it may affect viral transport, cleavage, and fusion. Previous studies have reported the H419R mutation within the F409-423 epitope in certain B3 and D8 wild-type MeV strains. Such mutations could potentially impair T cell recognition of F protein epitopes, thereby diminishing immune responses in vaccinated individuals.^20^ Future surveillance endeavors should center on whole - genome sequencing (WGS). Understanding the interaction among vaccine-induced immunity, WT-MeV strains, and mutational escape is of vital importance for effective measles control programs.

## 5 Conclusions

In this study, the N and F gene of MeV strains isolated in Shanghai over the past 21 years were sequenced, which was providing valuable insights into the genetic diversity and evolution of the virus. Specifically, 16 high-amino-acid-mutation sites in the H1a sub-genotype were identified. Additionally, most of the H1a and H1b strains exhibited a deletion of the glycosylation site at amino acids 9–11 (NVS). Despite these mutations, key functional sites in the F gene remained conserved, highlighting the gene's stability.

Ultimately, this study contributes to our understanding of measles virus genetics and emphasizes the need for sustained research and public health strategies to achieve measles control and elimination.

## CRediT authorship contribution statement

**Yunyi Li:** Writing – original draft, Formal analysis, Data curation, Conceptualization. **Xiaoxian Cui:** Data curation. **Ai Lin:** Writing – review & editing, Conceptualization. **Wei Tang:** Data curation. **Yuying Yang:** Methodology. **Wanju Zhang:** Software. **Jiayu Hu:** Investigation. **Zhi Li:** Investigation. **Yanqiu Zhou:** Writing – review & editing.

## Ethics statement

The throat swab samples used in this study were collected as a part of the National and Shanghai Municipal Measles and Rubella Surveillance Program and did not involve human experimentation. This study strictly followed the relevant ethical requirements and strictly protected the data security and privacy of the participants.

## Funding

The present study was supported by the key research project of Three-Year Initiative Plan for Strengthening Public Health System Construction in Shanghai (2023–2025) (GWVI-3), Three-Year Initiative Plan for Strengthening Public Health System Construction in Shanghai (2023–2025) Key Discipline Project (No. GWVI-11.1-12).

## Declaration of competing interest

We declare that there is no conflict of interest in our submission. All sources of funding for the research are disclosed. We have disclosed any personal or professional relationships that may be seen as presenting a potential conflict of interest with regards to the content of this manuscript. Additionally, any potential conflicts of interest that may arise in the future have also been disclosed.

## Data Availability

Data will be made available on request.
