# Supplementary material for: Genetic characterization on the nucleoprotein and fusion gene of wild-type measles virus circulating in Shanghai, 2001–2022
Source: J Virus Erad. 2025 Mar 5;11(1):100589. doi: 10.1016/j.jve.2025.100589 (PMC11930667; doi:10.1016/j.jve.2025.100589)
Supplement: Multimedia component 1 [file mmc1.docx]

**Supplementary Table 1**

**Table 1.** Wild-Type MeV isolates in Shanghai, 2001-2022

| Year | MeV isolates | Genotype／subgenotype | | | |
| --- | --- | --- | --- | --- | --- |
|  |  |  |  |  |  |
|  |  | H1a | H1b | D8 | B3 |
| 2001 | 15 | 11 | 4 | 0 | 0 |
| 2002 | 20 | 15 | 5 | 0 | 0 |
| 2003 | 52 | 51 | 1 | 0 | 0 |
| 2004 | 5 | 4 | 1 | 0 | 0 |
| 2005 | 23 | 22 | 1 | 0 | 0 |
| 2006 | 38 | 38 | 0 | 0 | 0 |
| 2007 | 24 | 24 | 0 | 0 | 0 |
| 2008 | 20 | 20 | 0 | 0 | 0 |
| 2009 | 9 | 9 | 0 | 0 | 0 |
| 2010 | 3 | 3 | 0 | 0 | 0 |
| 2011 | 37 | 37 | 0 | 0 | 0 |
| 2012 | 247 | 246 | 0 | 1 | 0 |
| 2013 | 338 | 336 | 0 | 0 | 2 |
| 2014 | 141 | 135 | 0 | 0 | 6 |
| 2015 | 372 | 372 | 0 | 0 | 0 |
| 2016 | 45 | 43 | 0 | 2 | 0 |
| 2017 | 8 | 8 | 0 | 0 | 0 |
| 2018 | 6 | 5 | 0 | 1 | 0 |
| 2019 | 2 | 0 | 0 | 2 | 0 |
| 2020 | 0 | 0 | 0 | 0 | 0 |
| 2021 | 0 | 0 | 0 | 0 | 0 |
| 2022 | 0 | 0 | 0 | 0 | 0 |
| Total | 1405 | 1379 | 12 | 6 | 8 |

**Table 2.** Amino acid substitution analysis of Shanghai Wild-Type MeV strains and S191 strains

| Strains | AA Position of N Protein | | | | | | | | | | | | | | | | | | | | | | | |
| --- | --- | --- | --- | --- | --- | --- | --- | --- | --- | --- | --- | --- | --- | --- | --- | --- | --- | --- | --- | --- | --- | --- | --- | --- |
|  | 406 | 422 | 427 | 431 | 441 | 443 | 447 | 448 | 450 | 451 | 455 | 456 | 457 | 462 | 467 | 470 | 471 | 473 | 481 | 482 | 484 | 497 | 505 | 509 |
| S191/A | I | G | N | R | K | S | A | R | S | Y | G | P | S | A | L | G | T | P | S | S | D | R | S | G |
| Cluster1-H1a | T | S | N | G | K | S | T | R | N | S | G | P | S | A | L | S | T | P | Y | S | E | R | L | G |
| Cluster2-H1a | T | G | N | G | K | S | T | R | N | S | G | P | G | A | L | S | T | P | Y | S | E | K | L | G |
| B3 | I | G | S | R | K | N | A | G | S | H | E | P | S | V | P | G | T | L | F | S | D | R | S | D |
| D8 | T | G | N | G | R | S | A | R | S | N | G | S | S | A | L | S | T | L | S | G | D | R | L | S |
